# Functional Nanogel from Natural Substances for Delivery of Doxorubicin †

**DOI:** 10.3390/polym14173694

**Published:** 2022-09-05

**Authors:** Katya Kamenova, Lyubomira Radeva, Krassimira Yoncheva, Filip Ublekov, Martin A. Ravutsov, Maya K. Marinova, Svilen P. Simeonov, Aleksander Forys, Barbara Trzebicka, Petar D. Petrov

**Affiliations:** 1 Institute of Polymers, Bulgarian Academy of Sciences, 1113 Sofia, Bulgaria; 2 Department of Pharmaceutical Technology and Biopharmaceutics, Faculty of Pharmacy, Medical University of Sofia, 1000 Sofia, Bulgaria; 3 Institute of Organic Chemistry with Centre of Phytochemistry, Bulgarian Academy of Sciences, 1113 Sofia, Bulgaria; 4 Research Institute for Medicines (iMed.ULisboa), Faculty of Pharmacy, Universidade de Lisboa, 1649-003 Lisbon, Portugal; 5 Centre of Polymer and Carbon Materials, Polish Academy of Sciences, 41-819 Zabrze, Poland

**Keywords:** nanogels, nanocarriers, citric acid, pentane-1,2,5-triol, doxorubicin, drug delivery

## Abstract

Nanogels (NGs) have attracted great attention because of their outstanding biocompatibility, biodegradability, very low toxicity, flexibility, and softness. NGs are characterized with a low and nonspecific interaction with blood proteins, meaning that they do not induce any immunological responses in the body. Due to these properties, NGs are considered promising candidates for pharmaceutical and biomedical application. In this work, we introduce the development of novel functional nanogel obtained from two naturally based products—citric acid (CA) and pentane-1,2,5-triol (PT). The nanogel was synthesized by precipitation esterification reaction of CA and PT in tetrahydrofuran using N-ethyl-N′-(3-dimethylaminopropyl) carbodiimide (EDC) and 4-(dimethylamino)pyridine (DMAP) catalyst system. Dynamic light scattering (DLS), cryogenic transmission electron microscopy (cryo-TEM) and atomic force microscopy (AFM) analyses revealed formation of spherical nanogel particles with a negative surface charge. Next, the nanogel was loaded with doxorubicin hydrochloride (DOX) by electrostatic interactions between carboxylic groups present in the nanogel and amino groups of DOX. The drug-loaded nanogel exhibited high encapsulation efficiency (EE~95%), and a bi-phasic release behavior. Embedding DOX into nanogel also stabilized the drug against photodegradation. The degradability of nanogel under acidic and neutral conditions with time was investigated as well.

## 1. Introduction

Nanogels are hydrogel particles formed by physically or chemically crosslinked three-dimensional polymer networks with dimensions typically between 10 and 200 nm [1]. They possess the typical highly hydrated nature and shrinking/swelling properties of hydrogels under different environmental conditions [2]. In recent years, nanogels have attracted great attention because of their outstanding biocompatibility, biodegradability, large specific surface area, very low toxicity, flexibility, and softness. They are characterized with a low and nonspecific interaction with blood proteins, meaning that they do not induce any immunological responses in the body. This fact makes nanogels promising candidates for pharmaceutical and biomedical applications. NGs have high stability, small size, high loading capacity and responsiveness to environmental factors, such as pH, temperature, and ionic strength, which is essential for modern nanomedicine. Their porous 3D structure allows the encapsulation of hydrophobic and/or hydrophilic drugs, potentially protecting the bioactive substances (BAS) from degradation during storage or blood circulation (e.g., hydrolysis or enzymolysis), and reduces toxic side effects. The loading of BAS in NGs can be achieved by physical interactions between the drug molecules and functional groups existing in NGs and usually happens spontaneously through hydrogen bonding, electrostatic, van der Waals and/or hydrophobic interactions. The physicochemical properties of NGs (surface charge, size, crosslinking density, and softness) can be adjusted by playing with the synthetic conditions, including monomer selection, monomer/crosslinker ratio, concentration, etc. [3,4,5,6].

Nanogels have been fabricated by polymerization of monomers (in a homogeneous phase, precipitation polymerization, micro-template polymerization, emulsion polymerization, emulsifier-free emulsion polymerization, dispersion polymerization, etc.) or by crosslinking of preformed polymers [6]. The most common method for the synthesis of NGs is the microemulsion polymerization in which the gel is obtained by combining an appropriate amount of water, oil, and surfactant(s). However, the nanogels synthesized in this way may contain large amounts of organic solvents and surfactant residues that are difficult to separate from the system and can lead to toxic reactions [2]. Soap-free emulsion polymerization is one of the eco-friendly methods by which narrowly dispersed nanogels can be obtained without the addition of surfactant [7]. Unfortunately, this technology is difficult to implement in the industry because of low polymerization rates and poor stability of the obtained emulsion. Precipitation polymerization is an alternative to the emulsion polymerization, having the advantages of a homogeneous reaction mixture (the monomer(s), crosslinking agent and initiator are homogeneously dissolved in the reaction medium before the reaction). When the polymerization/crosslinking reaction begins, at a certain chain length, the generated phase becomes insoluble, and separates form the solvent to form polymer colloidal particles that are precursors of nanogels [8].

The use of natural precursors for preparing polymeric carriers, including nanogels, is a beneficial strategy for developing drug delivery systems which are non-toxic, biodegradable, and biocompatible. Indeed, NGs based on chitosan [9], alginate/keratin [10], and alginate/gelatin [11] have been synthesized and assessed as drug carriers. pH-responsive alginate nanogel carriers of the anti-cancer drug DOX, obtained through in situ crosslinking with ionic calcium under ultrasound, showed a significantly higher accumulative release at pH 5.0 than at pH 7.4 [12]. Gyawali et al. developed photo-cross-linkable nanogels from a biodegradable polymer template with intrinsic photoluminescence and high photostability [13]. These fluorescent nanogels displayed excellent biodegradability and cytocompatibility owing to the biocompatible monomers citric acid, maleic acid, L-cysteine, and poly(ethylene glycol). The nanogels were further surface-functionalized with biologically active RGD peptides and loaded with DOX, resulting in a pH-responsive system capable of releasing the drug in acidic pH, resembling tumor environments.

Citric acid is a safe, nontoxic, low cost, water-soluble, UV-resistant, and biocompatible multifunctional reagent and it is generally regarded as safe (GRAS) by the US Food and Drug Administration (FDA) [14]. Owing to its three carboxylic groups, CA has been used as a covalent crosslinker of cellulose derivatives [15]. Recently, the synthesis of highly elastic super-macroporous cryogels fabricated by thermally induced crosslinking of 2-hydroxyethylcellulose (HEC) with citric acid was reported [16]. The polymer network was formed at elevated temperature by successive reactions of CA-based anhydride intermediates with HEC hydroxyl groups in bulk. An alternative mechanism of forming ester bonds under mild conditions is the Steglich esterification reaction using 1-ethyl-3-(3-dimethylaminopropyl) carbodiimide hydrochloride (EDC). For instance, Steglich esterification has been exploited for preparing hydrogels from alginate, collagen, and chitosan [17,18,19].

Doxorubicin is an anthracycline that is an important drug for treating various types of tumors [20,21]. Some of the main limitations regarding the use of doxorubicin include cardiotoxicity, tumor cell resistance, hydrolytic and photolytic degradation. [22,23]. A prospective strategy to overcome such problems is the development of drug delivery systems that can protect DOX from environmental factors and to provide its controlled delivery to the tumor tissue [24,25,26]. In this paper, we describe the synthesis of novel nanogel from two natural products—citric acid and pentane-1,2,5-triol. Pentane-1,2,5-triol is a renewable chemical derived from the hydrogenation of furfural, a by-product of lignocellulose processing [27]. CA and PT were crosslinked by the Steglich esterification precipitation reaction in THF at room temperature with N-ethyl-N′-(3-dimethylaminopropyl) carbodiimide and 4-dimethyl aminopyridine as a catalyst system. To the best of our knowledge, this is the first report describing fabrication of nanogels from CA and PT. Particle size and shape, size distribution, and surface charge of the nanogel were determined by using DLS, AFM and cryo-TEM. Next, the nanogel was loaded with the model drug DOX via electrostatic interactions between carboxylic groups present in the carrier and amino groups of DOX. The drug-loading capacity, encapsulation efficiency, release profile, biodegradability of nanogel and protecting effect towards photodegradation of DOX were investigated as well.

## 2. Materials and Methods

### 2.1. Materials

Citric acid (99.5%, Sigma-Aldrich, FOT, Sofia, Bulgaria), 4-(dimethylamino)pyridine (99%, Sigma-Aldrich, FOT, Sofia, Bulgaria), N-(3-dimethylaminopropyl)-N′-ethylcarbodiimide hydrochloride (for synthesis, Sigma-Aldrich, FOT, Sofia, Bulgaria), Furfuryl alcohol (98%, Sigma Aldrich, FOT, Sofia, Bulgaria), and doxorubicin hydrochloride (98.0–102.0%, Sigma-Aldrich, FOT, Sofia, Bulgaria) were used as received. Tetrahydrofuran (HPLC grade, Fisher Chemical, Labimex, Sofia, Bulgaria) were stirred overnight over calcium hydride and distilled prior to use.

### 2.2. Synthesis of Pentane-1,2,5-triol

The synthesis of PT was described elsewhere [28]. Briefly, furfuryl alcohol (20 g), dissolved in acetonitrile (200 mL), catalyst TS-1 (2 g) and H_2_O_2_ (30 mL; 37%) were reacted at 40 °C for 5 h. After work up, 6-hydroxy-2H-pyran-3(6H)-one was isolated as pale-yellow oil that crystallized in the freezer (−20 °C). Next, 6-hydroxy-(2H)-pyran-3(6H)-one (12.59 g, 0.11 mol), EtOH (275 mL) and Pd/C (1.26 g, 10 wt.%) catalyst were allowed to react in H_2_ atmosphere (balloon) at RT for 5 h. The reaction mixture was then filtered through a pad of Celite^®^ and the solvent was removed under reduced pressure to give 5-hydroxy-4-oxopentanal as a pale-yellow oil. Finally, 5-hydroxy-4-oxopentanal (12 g, 0.103 mol) was dissolved in MeOH (200 mL), and NaBH_4_ (11.72 g, 0.310 mol) was added slowly into portions at 0 °C. The reaction mixture was then warmed up to room temperature and stirred for 24 h. After that time a solution of iPrOH/HCl was slowly added to the suspension up to pH 2 to decompose the boronic complexes, followed by addition of K_2_CO_3_ (sat. sol. in MeOH) to neutralize the unreacted acid. Successive filtration through filter paper and nylon membrane filter (pore size 0.45 μm, diam. 47 mm), followed by solvent removal under vacuum yielded the desired product.

### 2.3. Synthesis of Nanogel from Penthane-1,2,5-triol and Citric Acid

Penthane-1,2,5-triol (0.1 g, 0.8 mmol, 1 eq.) and citric acid (0.46 g, 2.4 mmol, 3 eq.) were dried by azeotropic distillations with toluene and dissolved in anhydrous tetrahydrofuran (15 mL) under inert atmosphere in a 50 mL three-neck flask equipped with a magnetic stirrer. Then, EDC (0.23 g, 1.2 mmol, 1.5 eq.) and DMAP (0.12 g, 0.6 mmol, 0.75 eq.) were added into the solution. The esterification reaction was carried out at room temperature by stirring the solution for 72 h under inert atmosphere. The resulting solution of nanogel was filtered and additionally purified by dialysis (3500 MWCO, Spectrum Labs, New Brunswick, NJ, USA) against deionized water for removing THF for 5 days. Finally, the product was freeze-dried to obtain light-yellow powder. The synthesis procedure was repeated three times (to assess the reproducibility), resulting in samples denoted as NG1, NG2 and NG3.

### 2.4. Hydrolytic Degradation

The hydrolytic degradation of nanogel was assessed by DLS measurements carried out in neutral (pH = 6.5, deionized water) and acidic medium (pH = 3, hydrochloric acid).

### 2.5. Drug Loading of Nanogel

The nanogel was loaded with doxorubicin by the incubation method. Doxorubicin was added to an aqueous nanogel dispersion (1 mg/mL) at drug/nanocarrier mass ratio = 1:8.5 and 1:10. The dispersion was gently stirred (700 rpm) for 2 h and filtered (0.45 μm). The concentration of the loaded drug was determined spectrophotometrically at λ = 480 nm (Thermo Fisher Scientific, Waltham, MA, USA) and calculated according to a standard curve (10–80 μg/mL, r = 0.9991).

The drug-loading degree (*LD*) and encapsulation efficiency (*EE*) were calculated using Equations (1) and (2), respectively:

(1) $$LD\left(\%\right)=\frac{Total\;mass\;of\;drug-free\;drug}{Mass\;of\;drug-loaded\;nanogel}\times 100$$

(2) $$EE\left(\%\right)=\frac{Total\;mass\;of\;drug-free\;drug}{Total\;mass\;of\;drug}\times 100$$

### 2.6. In Vitro Release Test

In vitro release study was performed by the dialysis method. The nanogel dispersion (3 mL) was introduced into a dialysis membrane (10,000 MWCO, Spectrum Labs, New Brunswick, NJ, USA), which was placed in a buffer phase (20 mL) at 37 °C. Two buffer media were used as acceptor phase—citrate (pH = 5.0) and phosphate buffer (pH = 7.4). Samples of 3 mL were withdrawn from the buffer phase at predetermined times (1, 2, 4, 6, 8 and 24 h), and the concentration of the released doxorubicin was determined spectrophotometrically as described above.

### 2.7. DOX Stability Studies

Aqueous doxorubicin solution and dispersion of DOX-loaded nanogels in equimolar concentrations (0.3 mg/mL) were placed in glass vials and exposed to UV-irradiation (Dymax 5000-EC″ UV equipment with a 400 W metal halide flood lamp) at a dose rate of 5.7 J/cm^2^.min. Aliquots from both samples were withdrawn at certain intervals and the concentration of doxorubicin was determined as described.

### 2.8. Methods

FTIR spectra of freeze-dried nanogels were recorded from 600 to 4000 cm^−1^ using an attenuated total reflection (ATR) spectrometer (IRAffinity-1, Shimadzu, Kyoto, Japan). The size of nanoparticles (hydrodynamic diameter, D_h_) was determined with Zetasizer NanoBrook 90Plus PALS instrument, equipped with a 35-mW red diode laser (λ = 640 nm) at a scattering angle of 90°. The zeta potential was determined by the phase analysis light scattering (PALS) method at a scattering angle of 15°. Sample concentration was = 1.0 g L^−1^, and each measurement was performed in triplicate. Differential scanning calorimetry (DSC) analysis was carried out under a nitrogen atmosphere using a Perkin Elmer Differential Scanning Calorimeter, DSC-7, within the temperature range of 40–250 °C, at a heating rate of 10 °C/min. The ultraviolet–visible absorption spectra were recorded on a UV-vis spectrophotometer (Thermo Fisher Scientific, Waltham, MA, USA) using quartz cells with a path length of 1 cm. Cryogenic transmission electron microscopy (cryo-TEM) images were obtained using a Tecnai F20 X TWIN microscope (FEI Company, Hillsboro, OR, USA) equipped with field emission gun, operating at an acceleration voltage of 200 kV. Images were recorded on the Gatan Rio 16 CMOS 4k camera (Gatan Inc., Pleasanton, CA, USA) and processed with Gatan Microscopy Suite (GMS) software (Gatan Inc., Pleasanton, CA, USA). Specimen preparation was done by vitrification of the aqueous solutions on grids with holey carbon film (Quantifoil R 2/2; Quantifoil Micro Tools GmbH, Großlöbichau, Germany). Prior to use, the grids were activated for 15 s in oxygen plasma using a Femto plasma cleaner (Diener Electronic, Ebhausen, Germany). Cryo-samples were prepared by applying a droplet (3 μL) of the suspension to the grid, blotting with filter paper and immediate freezing in liquid ethane using a fully automated blotting device Vitrobot Mark IV (Thermo Fisher Scientific, Waltham, MA, USA). After preparation, the vitrified specimens were kept under liquid nitrogen until they were inserted into a cryo-TEM-holder Gatan 626 (Gatan Inc., Pleasanton, CA, USA) and analyzed in the TEM at −178 °C. Atomic force microscopy (AFM) analyses were conducted using a Bruker NanoScope V9 Instrument operating at 1.00 Hz scan rate under ambient conditions. Then, 2 μL of filtered colloid solution (1 g L^−1^) was placed onto a freshly cleaned glass substrate and spin-casted at 2000 rpm for a minute. The measurements were performed in ScanAsyst (Peak Force Tapping) mode. Wide-angle X-ray diffraction (WAXD) patterns were obtained on a Bruker D8 Advance ECO diffractometer, operating at 40 kV and 25 mA in Bragg–Brentano geometry with Ni-filtered Cu K_α_ radiation and a LynxEye-XE detector over the 2θ range of 5–50°, with a scanning rate of 0.02° s^−1^.

## 3. Results and Discussion

### 3.1. Synthesis of Nanogel

**Figure 1:**
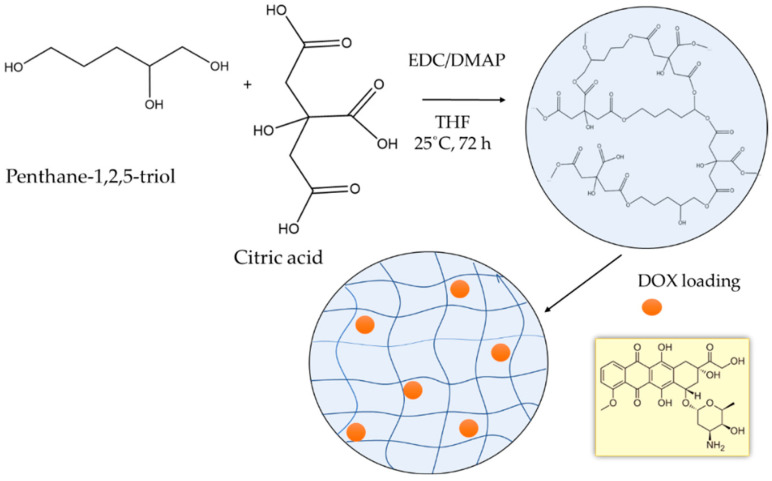
Preparation of DOX-loaded nanogel based on penthane-1,2,5-triol and citric acid.

Both penthane-1,2,5-triol and citric acid are naturally based polyfunctional reagents, which makes them attractive for fabricating new biomaterials. Moreover, the ester bonds formed after reacting CA with PT can be further hydrolyzed under given environmental conditions and, therefore, such materials are considered biodegradable. In this work, novel nanogel based on penthane-1,2,5-triol and citric acid was obtained at mild conditions via Steglich esterification precipitation reaction in THF. The formation of polymer nanonetwork proceeded at room temperature with the aid of N-ethyl-N′-(3-dimethylaminopropyl) carbodiimide as a coupling reagent and 4-(dimethylamino)-pyridine as a catalyst. Initially, all reagents (CA, PT, EDC and DMAP) were homogeneously dissolved in THF. Hence, the esterification crosslinking reaction started in a solution, but at a certain time, the formed polymeric phase became insoluble in THF and tended to precipitate. The generated insoluble particles were separated, purified, and dispersed in deionized water to form the nanogel. The presence of free a carboxylic group in the nanogel was further exploited for drug loading. The nanogel was loaded with the antitumor agent doxorubicin hydrochloride by electrostatic interactions between the carboxylic groups and amino groups of DOX. The scheme of preparing nanogel carriers of doxorubicin is shown in Figure 1.

**Figure 2:**
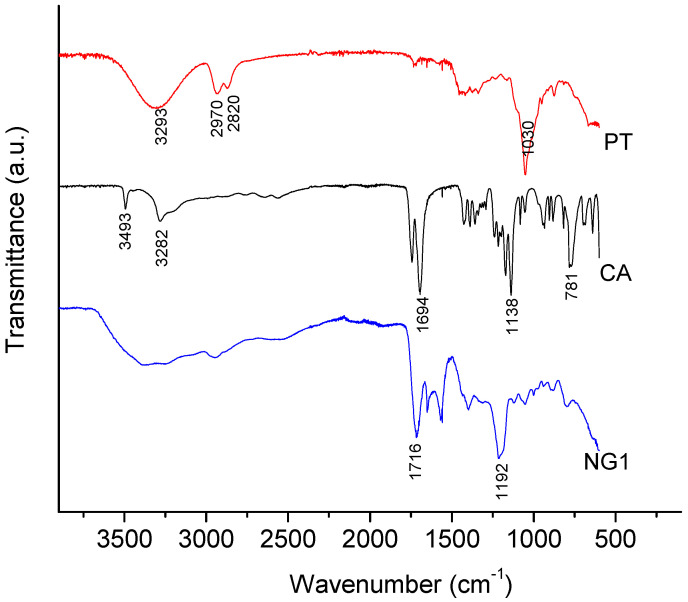
FTIR spectra of penthane-1,2,5-triol, citric acid and the nanogel (NG1) obtained on their basis.

Fourier-transform infrared spectroscopy was used in our study to gain an idea about the formation of ester bonds within the polymer network. Figure 2 shows the FTIR spectra of PA, CA and the nanogel formed on their basis. PT is characterized with the stretching vibration of the three -OH groups in the 3200–3500 cm^−1^ range; C-H stretching at 2820 and 2970 cm^−^^1^; and C-O stretching of the primary alcohol at 1030 cm^−1^ [29]. The most characteristic bands in the infrared spectra of CA are the O-H and C-H stretching vibrations in the 3500–3000 cm^−1^ range; C=O stretching (C(O)-OH) at 1694 cm^−1^; C-OH stretching at 1138 cm^−1^; and CH_2_ rocking at 781 cm^−1^ [30]. Two new intensive bands appeared in the FTIR spectrum of the nanogel—stretching vibrations of C=O groups at 1726 cm^−1^ and twisting vibration of CH_2_ groups at 1192 cm^−1^. These two bands are typical for polyesters [31]. Although the absorption intensity of the bands associated with -OH and -COOH groups was lower, the fact that they did not disappear completely suggests existence of free functional groups in the nanogel.

### 3.2. Properties of Nanogel

The surfactant-free synthesis of nanogel was repeated three times at the same conditions to assess the reproducibility of the method. The main characteristics of purified nanogel systems were determined by dynamic light scattering and zeta potential measurements (Table 1). Nanogels exhibited monomodal particle size distribution, nanoscopic hydrodynamic diameter and relatively narrow dispersity index (DI). Zeta potential values were negative, most probably due to the presence of -COOH groups within the polymer network.

**Figure 3:**
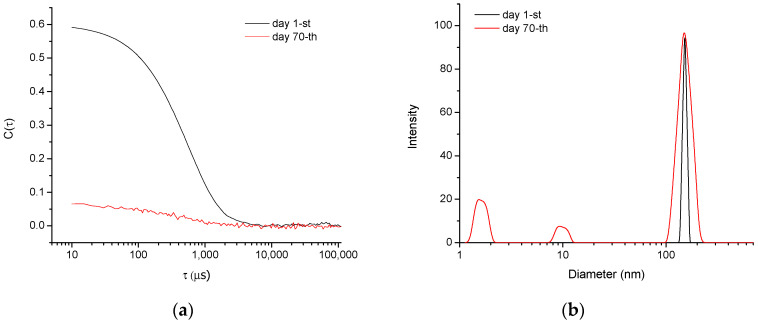
Autocorrelation function (**a**) and hydrodynamic diameter distribution (**b**) plots of NG1 in water (pH~6.5). Measurements were made on the first and seventieth day of sample preparation.

The structural stability of blank nanogels dispersed in deionized water (pH~6.5) over a prolonged period (3 months) was monitored by DLS (Figure 3 and Figure S1). Samples were measured every 10 days. In the first 30 days the nanogel dispersity index increased slightly. After that, the plot of the autocorrelation function tended to change its typical sigmoid shape and reached a nearly linear shape (after 70 days), while some smaller particles appeared in the intensity-weighted size distribution plot (Figure 3). The predominance of smaller fragments/monomers in the sample is evident from the number-weighted plot (Figure S1). These results indicated that the nanogel particles comprising ester bonds degrade in water with time. We hypothesize that the degradation mechanism follows the well-known reaction pathway of neutral hydrolysis of aliphatic polyesters, starting with nucleophilic attack of the labile ester bonds by water molecules [32]. In the beginning, the cleavage of ester links decreased the density of the polymer network, and the gel swelled more. Next, the network was gradually fragmented into smaller pieces, until its complete decomposition within 90 days (Figure S2). The process of gel degradation was much faster in an acidic environment. DLS results for nanogel dispersed in acidic water (pH = 3, HCl) revealed that the nanogel particles disappeared for a period of 20 days. The degradation under acidic conductions starts with protonation of the carbonyl oxygen atom of the ester group, which makes the carbonyl carbon atom more electrophilic. This facilitates the cleavage of C-O bonds of the main chain and accelerates the hydrolysis.

**Figure 4:**
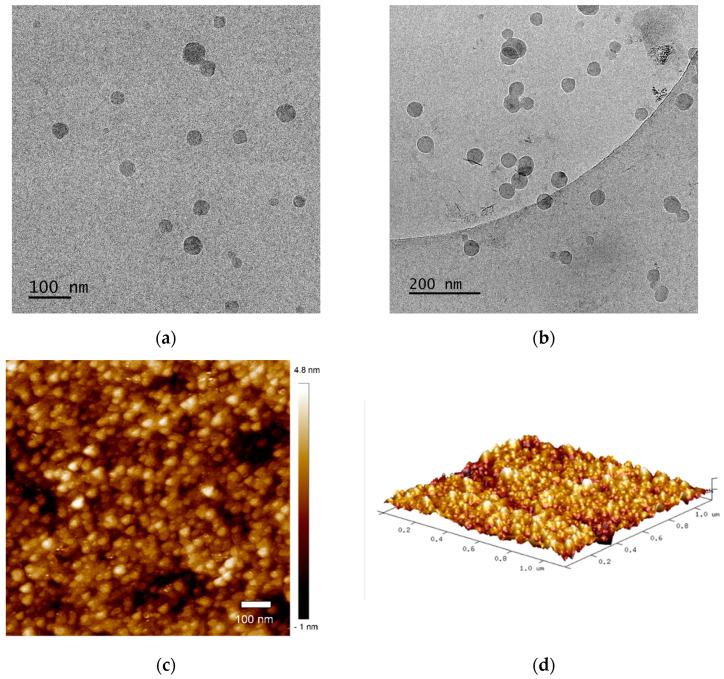
Representative cryo-TEM (**a**,**b**) macrographs and AFM 2D (**c**) and 3D (**d**) height images of NG1.

The morphology of nanogel particles (NG1) was visualized by cryogenic transmission electron microscopy and atomic force microscopy. Cryo-TEM micrographs of vitrified aqueous colloid (Figure 4a,b) showed that the main population of particles has a rather spherical shape and nanoscale size. However, some of the objects consist of two or three particles, most likely fused during the synthesis procedure. AFM images were obtained by spin-coating nanogel solution on a glass substrate and, therefore, represent the morphology of dry nanogel. In this case, the particles are smaller due to the dehydration of the gel, but the spherical shape is also well visible.

### 3.3. DOX-Loaded Nanogel

**Figure 5:**
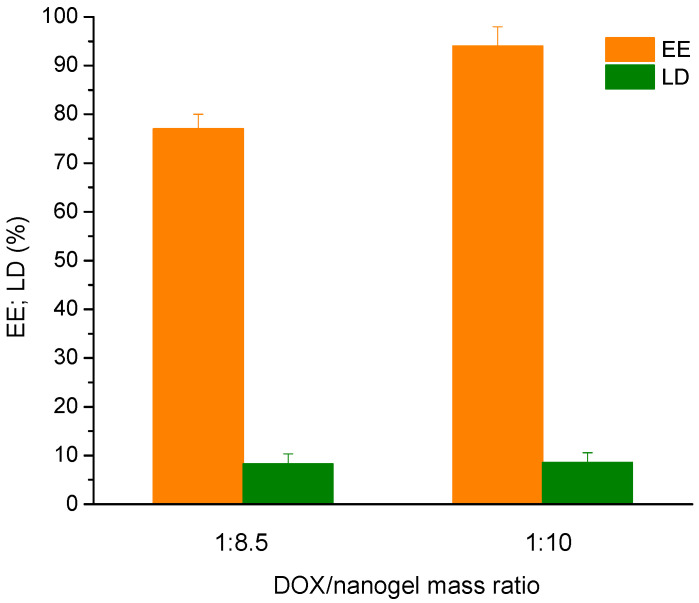
Drug-loading degree and encapsulation efficiency of doxorubicin in nanogel, prepared at mass ratio 1:8.5 and 1:10, respectively.

Loading of nanogel particles with the model drug doxorubicin was performed applying the incubation method. The expected driving force for embedding DOX molecules in the confined space of nanogel particles is the electrostatic interaction between carboxyl and amino groups from polymer network and drug, respectively. DOX-loaded nanogels maintained the initial size of blank carrier, but an increase of the dispersity and a decrease of zeta potential were recorded (Table 1). Such decrease of the surface charge of the nanogel is associated with carrier–DOX electrostatic interactions. This fact together with the increased solubility of DOX (a much smaller fraction of crystals was formed in the nanogel formulation compared to the free drug) confirmed that DOX was successfully embedded in the nanogel. The calculated encapsulation efficiency for the system obtained with 10-fold excess of polymer was higher (95%) than EE of the formulation prepared at a mass ratio 1:8.5 (Figure 5). In addition, the drug-loading degree of the two formulations was identical (~10%). Based on these results, DOX-loaded nanogel obtained at a mass ratio 1:10 was used in our further experiments.

**Figure 6:**
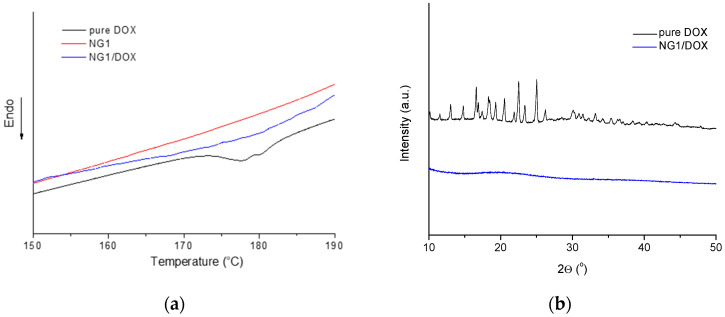
(**a**) DSC thermograms of pure doxorubicin, nanogel, and DOX-loaded nanogel, and (**b**) Wide-angle X-ray scattering spectra of pure doxorubicin and DOX-loaded nanogel.

DSC and WAXD analyses revealed that DOX embedded in the nanogel carrier is in amorphous state (Figure 6). The pure DOX is a crystalline substance which melted at about 178 °C (Figure 6a). As can be seen in the same figure, the melting peak was missing in the thermogram of DOX-loaded nanogel, confirming that the drug, loaded in the nanogel, did not form crystals. The XRD patterns of pure DOX exhibited many sharp diffraction peaks, ranging from 13–45°, indicating its crystalline structure (Figure 6b). In contrast, the pattern of lyophilized DOX-loaded nanogel is only a halo, confirming the amorphous state of DOX. The fact that DOX embedded in the nanocarrier is amorphous might be advantageous regarding its release behavior.

**Figure 7:**
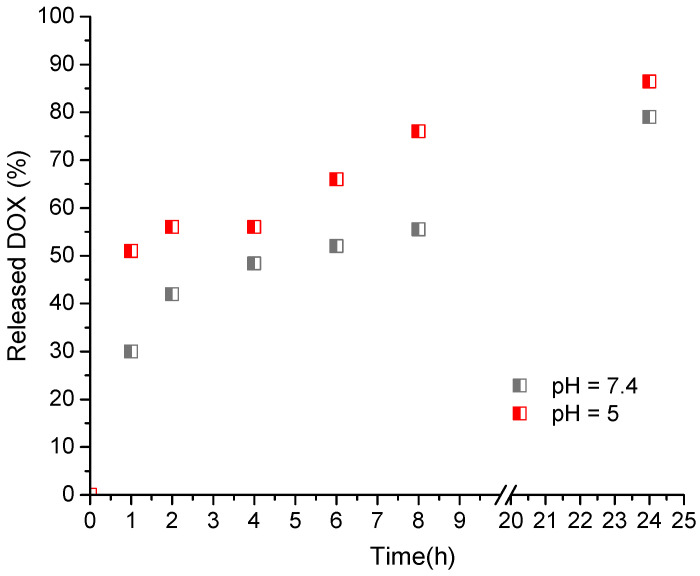
In vitro release of doxorubicin from the nanogel (NG1/DOX) in buffer media with pH-values 5.0 and 7.4.

In vitro release studies were performed in buffer media with pH values of 7.4 and 5.0, which resemble the slightly alkaline pH of most body fluids and the acidic pH of intracellular endosomal and lysosomal compartments, respectively. The release studies showed bi-phasic profiles (an initial burst release and then a gradual sustained release) of DOX in the two buffers (Figure 7). Kinetics analysis of the process revealed that the release followed the first-order model (Figure S3). This pattern describes the release of water-soluble drugs from porous matrices, suggesting formation of pores in the nanogel particles.

The burst effect was less pronounced in the medium with pH 7.4. In particular, 30% of doxorubicin was released for 1 h in the slightly alkaline medium vs. 51% in the acidic medium. Furthermore, the release rate in the acidic medium was higher than in the neutral one. Since DOX has a pH-dependent solubility we have performed a dissolution test of free DOX under the same conditions. The results revealed that at the applied concentration (equal to that of the encapsulated DOX) the drug was rapidly dissolved in both media for less than 30 min. The latter indicated that the reason for the observed different release profiles was the influence of the developed nanogel. A similar pattern of release profile of doxorubicin from pH-sensitive nanogels was reported by Jayakumar et al. [33]. It is attributed to the fact that in acidic conditions the amino groups of doxorubicin and the carboxyl groups of nanogel are protonated, which weakens the interaction between the drug and nanocarrier and leads to a fast drug release [34]. Drug delivery systems possessing such behavior might be beneficial in cancer therapy; however, more in vitro and in vivo studies are needed to assess the full capacity of the NG/DOX system for solid tumor curing.

**Figure 8:**
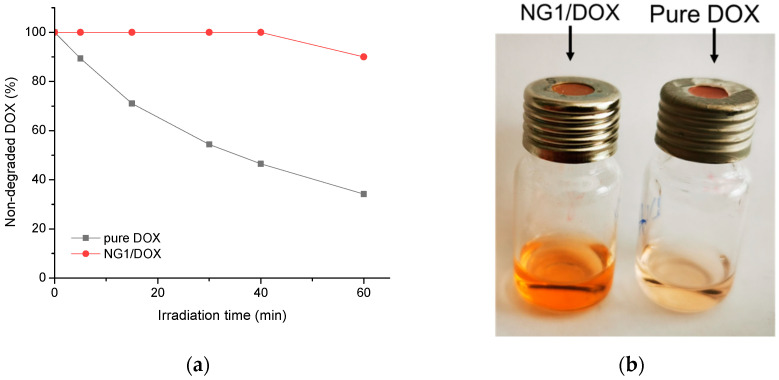
(**a**) UV-induced degradation of doxorubicin as a function of the irradiation time in DOX-loaded nanogel dispersion and pure DOX aqueous solution, and (**b**) digital image of the two samples after 60 min irradiation with UV light.

The low stability of doxorubicin in aqueous media (mainly hydrolytic and photolytic degradation) can be a limitation for its use in practice. For instance, Prokopowicz et al., observed a gradual irreversible photodegradation of doxorubicin in solution after only 1 h of exposure to UV–vis light, while the encapsulation of doxorubicin in a solid gel matrix prolonged the time of its use [35]. Recently, some of us reported that the formulation of DOX in appropriate drug delivery systems allows us to avoid the formation of degradation derivatives [25]. In the present work, we assumed that the inclusion of doxorubicin in nanogels can have a protective effect against light-induced degradation. Thus, the DOX-loaded nanogel and a referent DOX solution were exposed to UV-irradiation and the concentration of non-degraded drugs in both formulations was evaluated with time by spectrophotometry. The results showed a well pronounced decrease of doxorubicin concentration in the referent solution with the time, whereas DOX loaded into nanogel remained stable for 40 min (Figure 8). In fact, at the end of the experiment (60 min) 10% of the encapsulated DOX and 66% of the free DOX were degraded. This result is direct proof that the loading of doxorubicin into nanogel stabilized the drug against photodegradation.

## 4. Conclusions

Novel functional nanogel carriers of doxorubicin, based on the natural reagent citric acid and pentane-1,2,5-triol, were synthesized at mild conditions by nanoprecipitation esterification reaction without any surfactant. The synthesis procedure afforded fabrication of nano-sized gels of monomodal particle size distribution, negative zeta potential and relatively low dispersity index, in a reproducible way. The resulted nanogel particles were considered as an advantageous drug delivery system due to their biodegradability, high encapsulation capacity for doxorubicin and efficient drug protection against photolysis. Furthermore, the nanogel exhibited faster drug release in an acidic medium than in a neutral medium.

## Figures and Tables

**Table 1:** Data from dynamic light scattering and PALS measurements of nanogels.

Sample Code	D_h_ (nm)	ζ-Potential (mV)	DI
NG1	153 ± 4	−13.0 ± 1.2	0.22 ± 0.017
NG2	172 ± 5	−12.8 ± 1.1	0.23 ± 0.015
NG3	173 ± 5	−13.2 ± 1.2	0.32 ± 0.019
NG1/DOX	146 ± 4	−8.9 ± 1.0	0.40 ± 0.020

## Data Availability

Not applicable.

## Supplementary Materials

The following supporting information can be downloaded at: https://www.mdpi.com/article/10.3390/polym14173694/s1, Figure S1: Hydrodynamic diameter distribution plot of NG1 in water (pH~6.5); Figure S2: Schematic representation of the hydrolysis of nanogel in aqueous media; Figure S3: Kinetics analysis of DOX release from NG1 in buffer media with pH-values 5.0 and 7.4.
