# Supplementary material for: Functional Nanogel from Natural Substances for Delivery of Doxorubicin
Source: Polymers (Basel). 2022 Sep 5;14(17):3694. doi: 10.3390/polym14173694 (PMC9459996; doi:10.3390/polym14173694)
Supplement: Supplementary file 1 [file polymers-14-03694-s001.zip › polymers-1886844-supplementary.pdf]

polymers

MDPI

Supplementary Materials

Functional Nanogel from Natural Substances for Delivery of Doxorubicin †

Katya Kamenova [1], Lyubomira Radeva [2], Krassimira Yoncheva [2], Filip Ublekov [1], Martin A. Ravutsov [3], Maya K. Marinova [3], Svilen P. Simeonov [3,4], Aleksander Forys [5], Barbara Trzebicka [5] and Petar D. Petrov [1,*]

1 Institute of Polymers, Bulgarian Academy of Sciences, 1113 Sofia, Bulgaria
2 Department of Pharmaceutical Technology and Biopharmaceutics, Faculty of Pharmacy, Medical University of Sofia, 1000 Sofia, Bulgaria
3 Institute of Organic Chemistry with Centre of Phytochemistry, Bulgarian Academy of Sciences, 1113 Sofia, Bulgaria
4 Research Institute for Medicines (iMed.ULisboa), Faculty of Pharmacy, Universidade de Lisboa, 1649-003 Lisbon, Portugal
5 Centre of Polymer and Carbon Materials, Polish Academy of Sciences, 41-819 Zabrze, Poland
* Correspondence: ppetrov@polymer.bas.bg; Tel.: +359-2-9796335
† In Memory of Professor Andrzej Dworak.

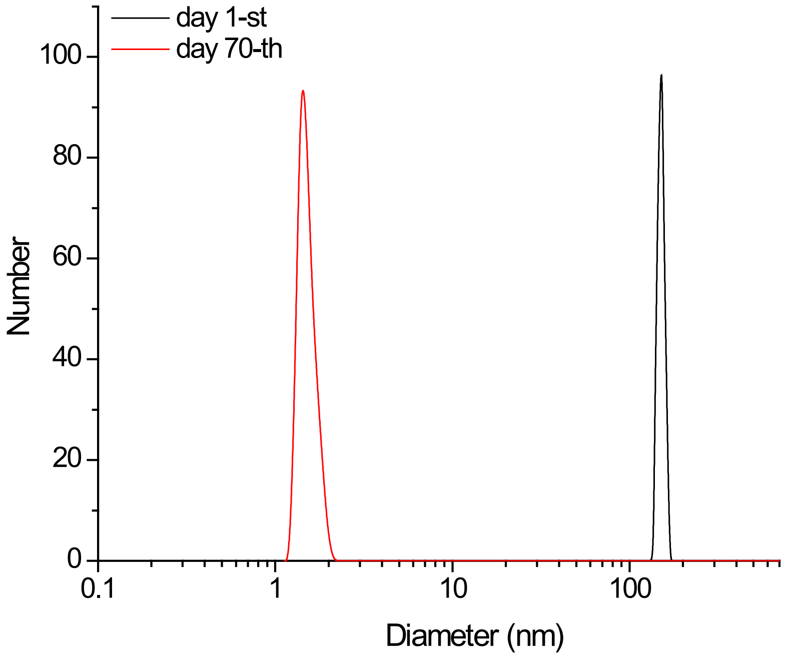

Figure S1. Hydrodynamic diameter distribution plot of NG1 in water (pH ~ 6.5). Measurements were made on the first and seventieth day of sample preparation.

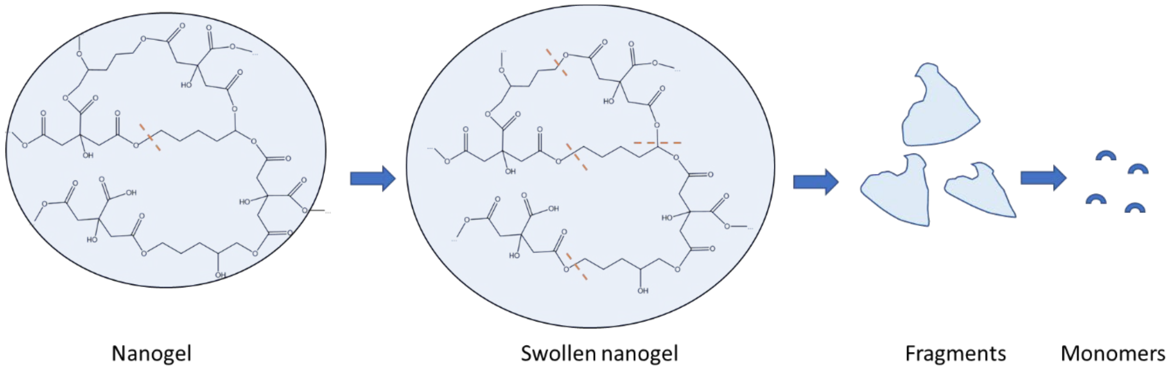

Figure S2. Schematic representation of the hydrolysis of nanogel in aqueous media.

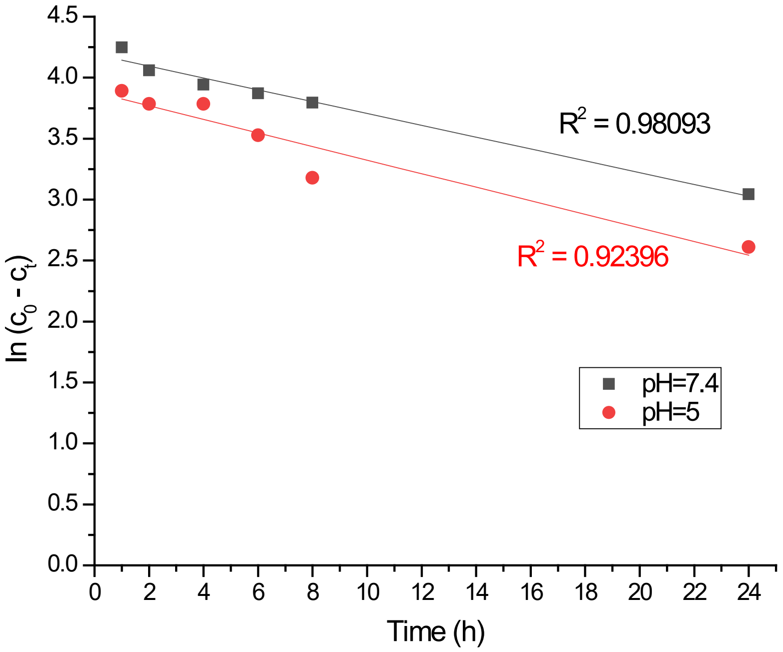

Figure S3. Kinetics analysis of DOX release from NG1 in buffer media with pH-values 5.0 and 7.4.
